# Case report—Stumped: back pain in a cricketer

**DOI:** 10.17159/2078-516X/2026/v38i1a26132

**Published:** 2026-08-15

**Authors:** L Lamote, JH Kirby

**Affiliations:** 1 Division of Sport and Exercise Medicine, Department of Exercise, Sport and Lifestyle Medicine, Faculty of Medicine and Health Sciences, Stellenbosch University, South Africa; 2 Campus Health Service, Stellenbosch University, South Africa; 3 KU Leuven, Department of Physical Medicine and Rehabilitation, Leuven, Belgium

**Keywords:** spondylolysis, lumbar bone stress injury, sacroiliitis, imaging, cricket

## Abstract

A 19-year-old collegiate fast bowler presented with chronic low back pain that acutely worsened during a tournament. Examination suggested a mechanical origin, but plain radiographs showed subtle lucency at L5 and bilateral sacroiliac sclerosis. MRI was interpreted as bilateral sacroiliitis with effusion, raising concern for axial spondyloarthropathy. Laboratory tests, including HLA-B27, were unremarkable, and rheumatological evaluation did not support a seronegative spondyloarthropathy. Computed tomography ultimately demonstrated stress fractures with developing spondylolysis at L4 and L5. The player was managed conservatively with physiotherapy, core strengthening, and a phased return to sport, including modification of his bowling technique. Full return to sport was achieved at six months after initial presentation. This case highlights the diagnostic pitfalls of interpreting MRI findings in young athletes, where mechanical changes may mimic inflammatory sacroiliitis. CT remains a valuable adjunct in detecting lumbar stress fractures when MRI findings are equivocal.

A 19-year-old male cricket fast bowler presented two months after an episode of acute on chronic low back pain (LBP). The exacerbation started during a tournament where he played daily for a week and slipped. Some lumbar discomfort described as stiffness during and shortly after activities had been present at a lower intensity for a year. Switching from bowling to batting did not resolve the pain, nor did physiotherapy. LBP was mostly present with high-intensity shots and when batting for over 20 minutes, was equally present in back- and front-foot shots and was situated in the same area as when bowling, but with greater severity. The patient reported 4/10 lumbar pain when picking up objects, situated deeply and centrally with radiation to both buttocks. It was aggravated by bowling and relieved by lying flat. He had no burning or shooting pain, and no numbness, sensory changes or weakness. He was avoiding lifting heavy objects. He was experiencing nocturnal pain that disturbed his sleep, but no night sweats, fever, weight loss or morning stiffness were present. There was no personal or family history of autoimmune diseases, and no comorbidities.

On clinical examination, there was discomfort on both active and passive range of motion in flexion, extension, lateral flexion and rotation. Dermatomes and myotomes were intact, and signs of radicular irritation were absent. Combined flexion, abduction and external rotation (FABER) of the hip provoked low back/sacroiliac joint (SIJ) pain bilaterally. The lumbar quadrant test was painful bilaterally on the ipsilateral side. The single-leg hop test was negative.

There was tender palpation of L5 at the midline, and palpation of the upper sacrum caused radiating pain to the lumbar spine on both sides. Extensive tinea versicolor of the skin was present, with no features of psoriasis. The clinical differential diagnosis included lumbar bone stress injury or facet joint irritation, with SIJ, malignancy or rheumatological causes as less likely possibilities.

Plain film radiographs showed sclerosis and subtle lucency at the L5 laminae without a typical Scotty dog presentation or spondylolisthesis. The SIJs showed bilateral, symmetric linear sclerosis and irregularity. Blood tests revealed an increased eosinophil count of 0.44 × 109/l; otherwise, the full blood count was normal. Erythrocyte sedimentation rate (ESR) was 7 mm/hr, C-reactive protein (CRP) was <1 mg/l, and HLA-B27 was negative. MRI was initially protocolled as bilateral sacroiliitis with sclerosis, fibrofatty metaplasia and right sacroiliac joint effusion, without features of an L5/S1 stress fracture or active sacroiliitis. A rheumatologist’s opinion was sought; axial spondyloarthropathy (axSpA) was deemed unlikely. A computed tomography (CT) scan was advised to better visualise the bony aspects of the lumbar region. CT imaging revealed incomplete stress fractures at L4 on the left and L5 bilaterally.

During the work-up, all cricket training was halted, and physiotherapy focused on restoring functional capacity for daily activities. Anti-inflammatories for two weeks had no effect. Once the diagnosis of stress fractures was made, management consisted of a cessation of sport in addition to core stability training, followed by a gradual return to training. He has since returned to bowling.

## Discussion

The lumbar region is one of the most common sites of injury among cricket players. Lumbar stress fractures accounted for up to 15% of all missed playing time in a cohort of Australian players, with the highest prevalence reported in cricket fast bowlers (24–55%).^[1]^ Specifically, players with a mixed bowling technique are more susceptible to bony lumbar stress injuries.^[1,2]^ Generally, the differential diagnosis of recurrent LBP in athletes includes facet joint syndrome, herniated disc with dural or radicular irritation, non-specific mechanical LBP, lumbar bone stress injury (LBSI), axSpA, and, in rare cases, malignancy. Spondylolysis has been widely used in literature as an umbrella term for LBSI, including lumbar stress reactions, lumbar stress fractures and chronic non-union defects after stress fractures, making it difficult to quantify the exact prevalence of respective conditions. These injuries present a continuum from bone stress reaction to fracture; the term LBSI is more relevant today.

In our case, given the mechanical character of the symptoms and the increased workload and repetitive lumbar loading during fast bowling, LBSI was at the top of the differential diagnosis. Repetitive overhead throwing motions associated with fast bowling and the clinical findings of pain at combined extension and rotation supported this. We opted for an X-ray as the first imaging modality due to its accessibility and low cost.^[3]^ Additionally, our radiology department does not perform an MRI without a prior plain film X-ray. Limitations of plain radiography include a low sensitivity for early/stress lesions and the inability to stage lesion activity.^[3]^ Surprisingly, bilateral sclerosis of the SIJs was described in the absence of a lumbar stress fracture, with only a subtle lucency present at the L5 laminae. Further investigation was required to determine whether a stress fracture or reaction was present and whether sacroiliac sclerosis was the result of inflammatory joint activity or mechanical load. Considering his young age, axial SpA needed to be excluded. Other less probable causes of symmetrical SIJ sclerosis in athletes include osteitis condensans ilii, an accessory sacroiliac joint, chronic infection, autoinflammatory osteitis, diffuse idiopathic skeletal hyperostosis (DISH) and disorders of mineral metabolism.^[4]^

**Fig. 1:**
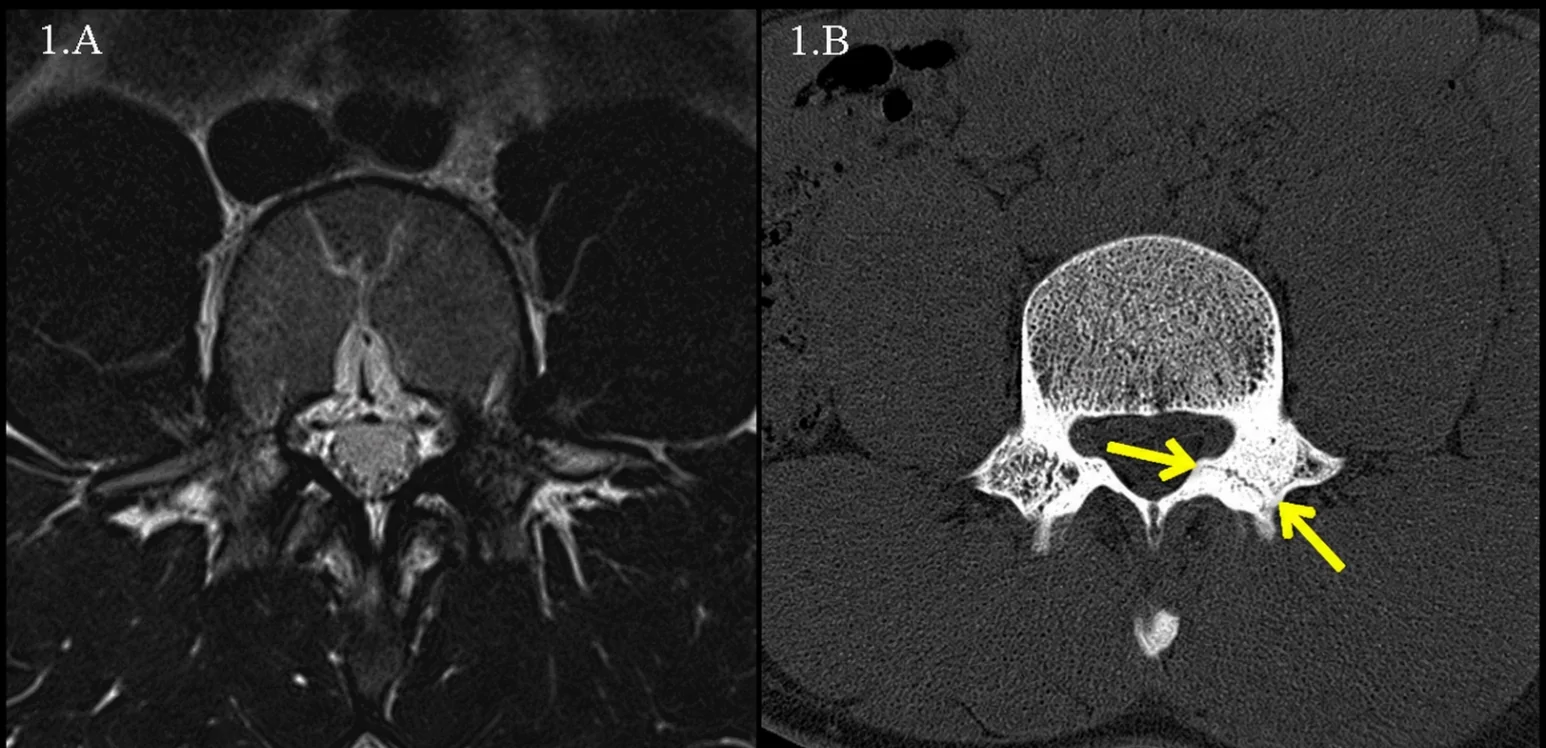
A: Axial T2 MRI sequence of L4 vertebra; B: Axial CT sequence of L4 vertebra. Yellow arrow indicates stress fracture. CT, computed tomography; L4, fourth lumbar vertebra; MRI, magnetic resonance imaging.

MRI shows excellent sensitivity for detecting bone marrow oedema and was the preferred imaging technique for further investigation, as sacroiliitis is a criterion for the diagnosis of axial SpA. By contrast, its limitations include financial cost and its sensitivity for fracture detection, which has been reported to be lower than that of CT. However, advances in imaging techniques have shown excellent sensitivity (97%), specificity (100%), and accuracy (97%) for detecting any type of fracture, complete or incomplete, compared with CT in recent studies using 3 Tesla T1-weighted volumetric interpolated breath-hold imaging.^[3,5]^ In our patient, the MRI was initially reported as showing features of bilateral sacroiliitis with sclerosis but with insufficient evidence for active sacroiliitis, which requires bone marrow oedema, synovitis, capsulitis or enthesitis.^[6]^ No features of bone stress were reported on the L5 vertebral body or pars interarticularis (Figure 1A). The above radiological findings could be associated with SpA and chronic sacroiliitis. As the blood tests, including HLA-B27, did not indicate an inflammatory disease, the patient was referred to a rheumatologist to exclude seronegative SpA.

According to the ASAS criteria, a diagnosis of axial SpA requires sacroiliitis on imaging with at least one SpA feature or an elevated serum HLA-B27 in the presence of at least two SpA features.^[6]^ After rheumatological evaluation, and in the absence of any SpA feature except for chronic sacroiliitis on imaging, a diagnosis of seronegative SpA was unlikely. It was judged that no convincing features of spondyloarthritis, e.g., episodes of peripheral enthesitis or systemic features such as uveitis, psoriasis, urethritis, or enterocolitis, were present. Furthermore, the pain was mechanical in character because the LBP started with a period linked to higher activity loads, exacerbating after a traumatic event, and worsening with activity, whereas an inflammatory backache has an insidious onset and usually improves with activity.

The SIJ changes in our patient likely represent an adaptation to physical strain, rather than an immune-mediated inflammation. The location of subchondral bone marrow oedema is a crucial factor in distinguishing between mechanical changes and sacroiliitis. The former typically occurs in the anterior part of the mid-third of the SIJ, where mechanical stress is concentrated.^[4]^ The diagnostic pathway was influenced by the incidental finding of bilateral SIJ sclerosis on radiographs and the initially described MRI findings, prompting further evaluation for axial spondyloarthritis despite the absence of anamnestic or biochemical SpA features. A lumbar bone stress injury remained more likely given the patient's fast-bowling workload and clinical presentation. Review of the initial MRI revealed bone marrow oedema at the L5 lamina, suggestive of a stress reaction. Additional CT imaging confirmed a stress fracture at the L4 and L5 levels, highlighting the advantage of CT in detecting bony lesions (Figure 1B). A possible explanation for the discrepancy between the two imaging modalities is that, in our patient, MRI sequences included T1, T2 and STIR sequences at 1.5-T in contrast with 3-T techniques used in recent studies comparing CT to MRI.^[5]^

A complete cessation of sport was implemented for one month, after which a core-strengthening program was added. A modified return was achieved at three months. His bowling technique was adapted from a mixed to side-on technique, and he used a shortened run-up. A full return to bowling was achieved at six months after the initial cessation of sport.

Our case illustrates the pitfalls in interpreting sacroiliitis on MRI and underscores the importance of clinical judgement in the diagnosis of axSpA. Although new MRI techniques show excellent sensitivity and reliability for detecting stress reactions and fractures, one should be aware that some osseous lesions can be missed. In these cases, additional CT imaging is useful to detect fractures, especially in individuals at risk of developing overuse lumbar injuries in the presence of mechanical LBP.

## Conclusion

This case illustrates the diagnostic complexity of low back pain in young athletes, as mechanical stress-related changes can resemble sacroiliitis on imaging. While radiographic and MRI findings suggested axSpA, the clinical presentation was indicative of lumbar bone stress injury. CT confirmed incomplete pars stress fractures at L4 and L5, demonstrating its utility when MRI findings and the clinical picture appeared discordant. Awareness of these diagnostic challenges is essential to avoid misdiagnosis, guide appropriate imaging strategies, and facilitate safe return to sport.
